# Model Amphipathic Peptide Coupled with Tacrine to Improve Its Antiproliferative Activity

**DOI:** 10.3390/ijms22010242

**Published:** 2020-12-29

**Authors:** Sara Silva, Cláudia Alves, Diana Duarte, Ana Costa, Bruno Sarmento, António J. Almeida, Paula Gomes, Nuno Vale

**Affiliations:** 1 OncoPharma Research Group, Center for Health Technology and Services Research (CINTESIS), Rua Dr. Plácido da Costa, 4200-450 Porto, Portugal; saracpsilva21@gmail.com (S.S.); dianaduarte29@gmail.com (D.D.); 2 Faculty of Pharmacy, University of Porto, Rua de Jorge Viterbo Ferreira, 228, 4050-313 Porto, Portugal; 3 Research Institute for Medicines (iMed.ULisboa), Faculty of Pharmacy, Universidade de Lisboa, Avenida Professor Gama Pinto, 1649-003 Lisboa, Portugal; aalmeida@ff.ulisboa.pt; 4 Laboratório Associado para a Química Verde (LAQV), Network of Chemistry and Technology (REQUIMTE), Departamento de Química e Bioquímica, Faculdade de Ciências, Universidade do Porto, Rua do Campo Alegre, 687, 4169-007 Porto, Portugal; claudialves05@gmail.com (C.A.); pgomes@fc.up.pt (P.G.); 5 Instituto de Engenharia Biomédica (INEB), University of Porto, Rua Alfredo Allen, 208, 4200-135 Porto, Portugal; ana.margarida.costa@ineb.up.pt (A.C.); bruno.sarmento@ineb.up.pt (B.S.); 6 Instituto de Investigação e Inovação em Saúde (i3S), University of Porto, Rua Alfredo Allen, 208, 4200-135 Porto, Portugal; 7 Instituto de Investigação e Formação Avançada em Ciências e Tecnologias da Saúde & Instituto Universitário de Ciências da Saúde (CESPU), Rua Central de Gandra, 1317, 4585-116 Gandra, Portugal; 8 School of Pharmacy, Queen’s University Belfast, Medical Biology Centre, 97 Lisburn Rd, Belfast BT9 7BL, UK; 9 Faculty of Medicine, University of Porto, Al. Prof. Hernâni Monteiro, 4200-319 Porto, Portugal

**Keywords:** model amphipathic peptide, cell-penetrating peptides, tacrine, blood-brain barrier, MCF-7 cells, anticancer activity, SH-SY5Y cells

## Abstract

Drug repurposing and drug combination are two strategies that have been widely used to overcome the traditional development of new anticancer drugs. Several FDA-approved drugs for other indications have been tested and have demonstrated beneficial anticancer effects. In this connection, our research group recently reported that Tacrine, used to treat Alzheimer’s Disease, inhibits the growth of breast cancer MCF-7 cells both alone and in combination with a reference drug. In this view, we have now coupled Tacrine with the model amphipathic cell-penetrating peptide (CPP) MAP, to ascertain whether coupling of the CPP might enhance the drug’s antiproliferative properties. To this end, we synthesized MAP through solid-phase peptide synthesis, coupled it with Tacrine, and made a comparative evaluation of the parent drug, peptide, and the conjugate regarding their permeability across the blood-brain barrier (BBB), ability to inhibit acetylcholinesterase (AChE) in vitro, and antiproliferative activity on cancer cells. Both MAP and its Tacrine conjugate were highly toxic to MCF-7 and SH-SY5Y cells. In turn, BBB-permeability studies were inconclusive, and conjugation to the CPP led to a considerable loss of Tacrine function as an AChE inhibitor. Nonetheless, this work reinforces the potential of repurposing Tacrine for cancer and enhances the antiproliferative activity of this drug through its conjugation to a CPP.

## 1. Introduction

In 2018, cancer accounted for 9.6 million deaths worldwide and remains considered the second-leading cause of death [1]. For several years, the treatment of cancer has been based on tumor surgical removal (whenever possible), radiotherapy, and pharmacotherapy. However, successful treatment is still hard to achieve and conventional chemotherapy is associated with severe adverse effects on patients. Over the years, there has been an increased investment in the development of new compounds for cancer therapy. Still, only a few drugs have been successful to reach the clinics. As such, new strategies are required to efficiently develop more effective anti-cancer compounds.

**Figure 1:**
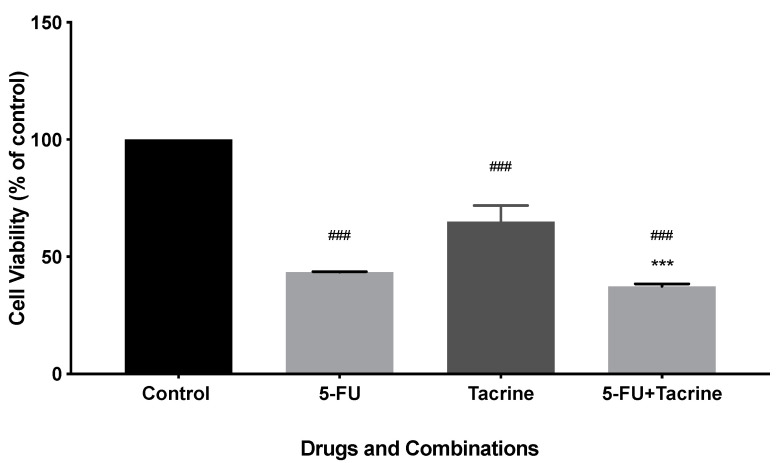
Effect of Tacrine and 5-Fluorouracil (5-FU) on the viability of MCF-7 cells. Results are presented as mean ± standard error of the mean (SEM) and represent the viability of cells (% of control) of three independent experiments (*n* = 3). ### *p* < 0.001 vs. control; and *** *p* < 0.001 vs. single drug of the combination with more effect on cell viability reduction.

Drug combination is a strategy increasingly used in oncology because it allows taking advantage of the maximum therapeutic effect of two or more drugs, by combining different mechanisms of action, while reducing required doses and, consequently, side effects. Drug repurposing is another strategy that allows overcoming the disadvantages of the traditional development of novel anticancer drugs, by searching for new therapeutic uses for drugs already approved for other indications. This allows the reduction of costs related to the development of new drugs while promoting a faster route towards the discovery of new therapies. Moreover, repurposed drugs have already been well characterized regarding their toxicity, dose efficacy, and plausible mechanism of action. Tacrine (Cognex^®^), the first-generation acetylcholinesterase (AChE) inhibitor approved by the U. S. Food and Administration (FDA) for the treatment of Alzheimer’s disease (AD) in 1993 [2,3], has already been tested on a variety of cancer cell lines, to assess its potential for repurposing to oncological pharmacotherapy [4,5,6,7,8]. Recently, our group also investigated the activity of Tacrine in breast tumor cells (MCF-7), both alone and in combination with 5-Fluorouracil (5-FU), in an attempt to improve the activity of this antineoplastic and reduce its therapeutic dose. The results demonstrated that Tacrine had antiproliferative activity at 50 µM, and its combination with 5-FU showed very promising results as it increased the cytotoxicity against those tumor cells (Figure 1) [8].

In view of the above, we decided to explore how the coupling of Tacrine with the cell-penetrating peptide (CPP) MAP might enhance its anti-cancer effect. CPPs are considered versatile carriers due to their unique ability to transport a wide range of molecules such as proteins, nucleic acids, contrast agents, drugs, and promote their intracellular delivery with limited toxicity [9]. Over recent years, a variety of new CPPs have been explored which displayed specific targeting properties towards cancer cell lines [10,11,12]. MAP (named after “model amphipathic peptide”) is an artificial CPP that integrates both hydrophobic and hydrophilic residues in opposite sides of its α-helical structure, resulting in a very well-defined amphipathic α-helix [13]. This CPP, whose sequence is KLALKLALKALKAALKLA, has the ability to translocate a variety of compounds such as oligonucleotides [14], peptides [15], and small proteins (like cytochrome c) across cell membranes [16]. Moreover, in a study by Hällbrink et al., addressing the cellular uptake kinetics of several CPP, MAP was found to display the fastest cellular uptake, along with the ability to induce leakage by disturbing the membrane integrity at concentrations as low as 1 μM [17].

The approach herein reported comprised the chemical synthesis of both the peptide MAP and its Tacrine conjugate, which was followed by a comparative assessment of (i) permeability on a well-known blood-brain barrier (BBB) model using hCMEC/D3 cells [18,19,20], and (ii) ability to inhibit AChE in vitro using the Ellman’s method [21], of the conjugate and its parent compounds, that is, MAP and Tacrine individually. Next, the antiproliferative activity of these compounds was assessed in vitro, on breast (MCF-7) and human neuroblastoma (SH-SY5Y) cancer cell lines [21,22,23], using the standard 3-(4,5-dimethyl-2-thiazolyl)-2,5-diphenyl-2H-tetrazolium bromide (MTT) reduction assay as well as evaluation of cellular morphological changes, to assess and compare the conjugate’s toxicity and membrane disruption characteristics, in both cells lines.

## 2. Results and Discussion

### 2.1. Chemical Synthesis of MAP and Its Tacrine Conjugate

**Figure 2:**
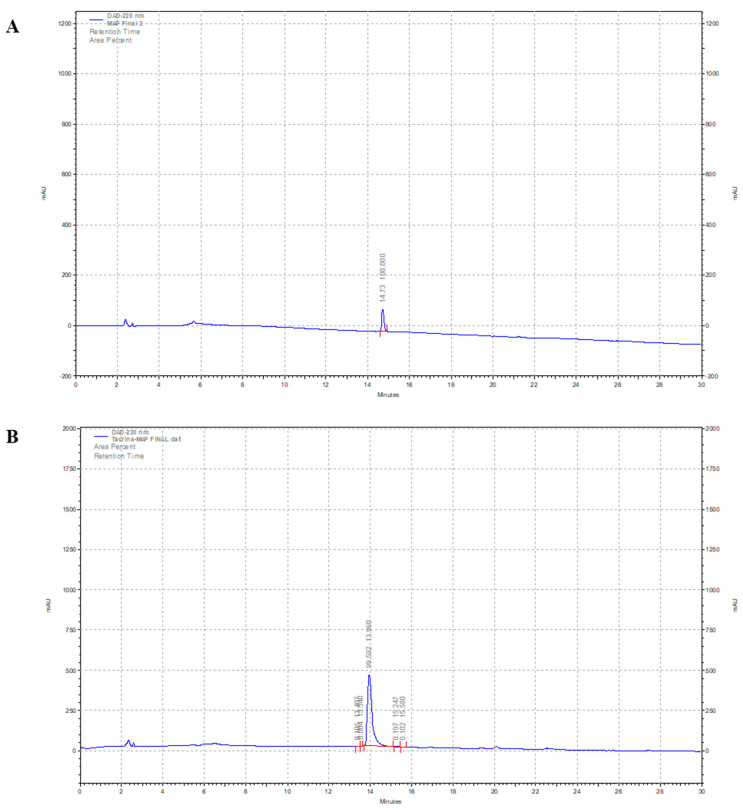
Chromatograms of synthetic MAP (**A**) and MAP-Tacrine conjugate (**B**), acquired with an HPLC system, with a C18 column, using ACN and acidified water (0.05% TFA) as eluent, in gradient mode (0–100%), for 30 min, at a flow rate of 1 mL/min and detection at λ = 220 nm.

Peptide MAP (KLALKLALKALKAALKLA-NH_2_) was synthesized through Fmoc/tBu solid-phase peptide synthesis (SPPS) methodologies, see Supplementary Information (SI), Figure S1. After cleavage and purification of the crude product, as given in the SI, the isolated peptide was analyzed by high-performance liquid chromatography (HPLC, Figure 2A and Figure S2) and liquid chromatography–mass spectrometry (LC-MS, Figure S3). HPLC analysis showed a distinct peak [retention time (r_t_) = 14.7 min] corresponding to a purity degree of 100%. The exact and observed (base peak) molecular mass of MAP obtained by LC-MS were 1876.50 and 1877.32 Da, respectively, which confirmed the successful synthesis of the target peptide as its quasi-molecular ion, [M + H]^+^. It was also possible to observe minor peaks corresponding to different protonation states of the peptide.

**Scheme 1:**
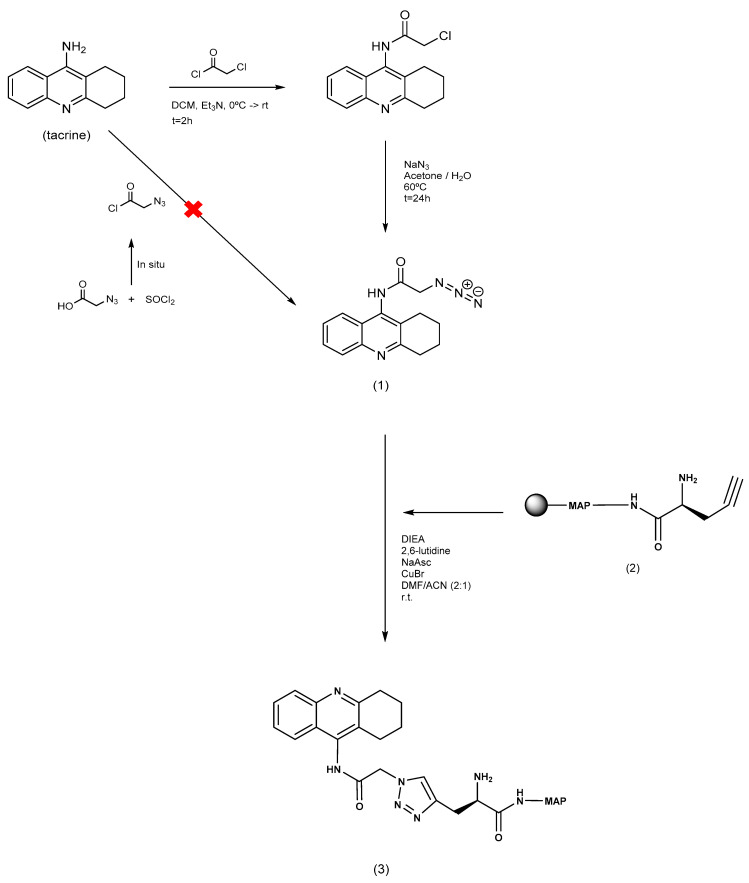
Representation of the methodology applied for the synthesis of the Tacrine-MAP conjugate by SPPS. Tacrine-MAP (**3**) was obtained with a high level of purity (99.6%). Red cross means that the reaction was executed but did not evaluated. Synthetic details, analytical and spectral data are supplied as Supplementary Information (SI, Figures S1–S15).

Additionally, an alkyne-modified version of MAP was synthesized (Pra-MAP, **2** in Scheme 1) through the same methods described above (see also SI), in order to allow the conjugation of the CPP to the drug. The isolated peptide was analyzed by HPLC (Figure S4) and matrix-assisted laser desorption/ionization (MALDI-TOF, Figure S5).

The subsequent conjugation was achieved through one of the best known “click chemistry” reactions, the classical copper-catalyzed azide−alkyne coupling (CuAAC) or Huisgen’s 1,3-dipolar cycloaddition (Scheme 1). CuAAC results in the chemoselective formation of a 1,2,3-triazole and can be carried out in either water or organic solvents under very mild conditions, while offering the possibility of being employed post-synthetically by use of purified units modified with alkynyl and azido functionalities, without need for additional protection of other functional groups. In this work, the CuAAC was promotedon-resin, between an azide-modified derivative of Tacrine (**1**, Scheme 1) and the alkyne-modified version of MAP (Pra-MAP, **2** in Scheme 1) in the presence of copper (I) bromide, diisopropylethylamine (DIEA), sodium ascorbate (NaAsc) and 2,6-lutidine, for 24 h at room temperature. Relevantly, the azide moiety could not be directly introduced in Tacrine through the reaction of its aniline group with azidoacetyl chloride, which was circumvented by first coupling chloroacetyl chloride onto tacrine, followed by conversion of the terminal chlorine into azide upon reaction with sodium azide (Scheme 1). After cleavage from the solid support and precipitation using *tert*-butylmethyl ether (<0 °C), the target MAP-Tacrine conjugate (**3** in Scheme 1) was purified on a preparative scale, under a suitable gradient of acidified H_2_O (0.05% trifluoroacetic acid, TFA) and acetonitrile (ACN).

The structure of the Tacrine derivative **1** was confirmed by electrospray ionization mass spectrometry (ESI-MS), ^1^H and ^13^C-NMR spectroscopy, whereas the peptide-drug conjugate **3** was characterized by HPLC and LC-MS, as given in detail in the SI (Figure 2B and Figures S11 and S12).

### 2.2. Permeability Assays

In order to evaluate the potential advantages of coupling MAP to Tacrine, as compared to Tacrine alone, we performed an in vitro BBB permeability study using hCMEC/D3 cells as a BBB model. This would not only allow determining whether peptide conjugation might cause loss of Tacrine’s BBB permeability, as well as establish if the conjugate might cause barrier dysfunction. Trans-epithelial electrical resistance (TEER) measurements were conducted in order to assess barrier integrity throughout every measuring point of the experiment. These values are the variance of the electrical resistance across time (Figure S16). As TEER values demonstrated a lack of barrier integrity right from the start of the experiment (Table S1) with 0 Ω/cm^2^ and high standard deviation (SD) values, the results of this experiment were inconclusive (Figure S16).

### 2.3. AChE In Vitro Inhibition Assay

**Figure 3:**
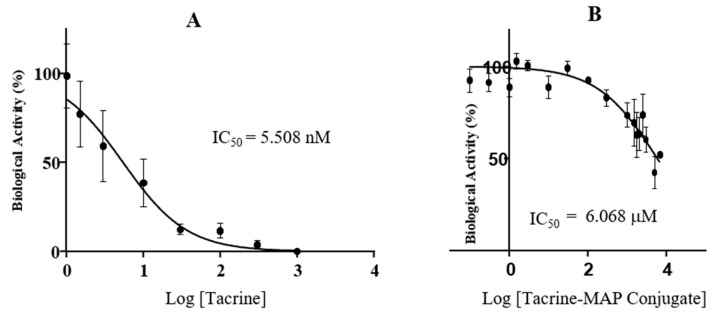
Log [drug] vs. biological activity (%) of Tacrine (**A**) and the conjugate (**B**). Results are presented in mean ± standard error of mean (SEM). The IC_50_ of each compound is displayed next to the inhibition curves.

Considering that Tacrine is a well-known AChE inhibitor, it was necessary to know if its coupling to the CPP could alter its AChE inhibition properties. After performing the Ellman’s method and calculating the IC_50_, we obtained a value of 5.508 nM for Tacrine (Figure 3A) and 6068 nM for the conjugate (Figure 3B), which clearly shows that the AChE inhibitory capacity of Tacrine is severely affected upon conjugation with MAP.

### 2.4. Toxicity of Tacrine and Its MAP Conjugate to MCF-7 and SH-SY5Y Cells

**Figure 4:**
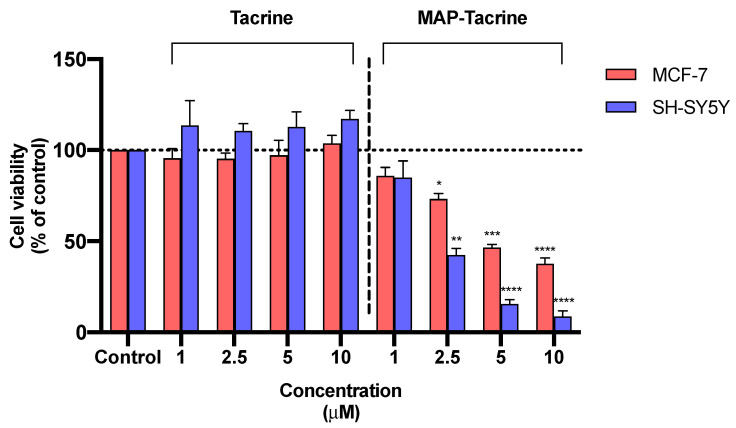
MCF-7 breast cancer and SH-SY5Y cell line cytotoxic effects of increasing concentrations of Tacrine, and Tacrine-MAP after a 24 h incubation. H_2_O was used in control. Significant differences (ANOVA test, followed by the with Dunnett’s post hoc test): * *p* < 0.05, ** *p* < 0.01, *** *p* < 0.001 and **** *p* < 0.0001 vs. Control. The values represent mean ± SEM (*n* = 3).

The toxicity of Tacrine and Tacrine-MAP towards MCF-7 (breast cancer) and SH-SY5Y (neuroblastoma) cells was tested at four different concentrations (1, 2.5, 5, and 10 µM), using the MTT reduction assay after a 24 h-incubation period. Results show no significant differences in MTT reduction by Tacrine alone, when compared to control, for both cell lines (Figure 4). On the other hand, the MAP-Tacrine conjugate caused a significant mitochondrial dysfunction for the three higher concentrations, as reflected by the decrease in the ability to metabolize MTT both by MCF-7 (2.5, 5 and 10 μM–(73.1 ± 4.4), (46.6 ± 2.4) and (37.7 ± 4.5)% of control cells, respectively) and, more pronouncedly, SH-SY5Y (2.5, 5 and 10 μM–(42.4 ± 5.2), (15.5 ± 3.4) and (8.7 ± 4.4)% of control cells, respectively) cells (Figure 4).

**Figure 5:**
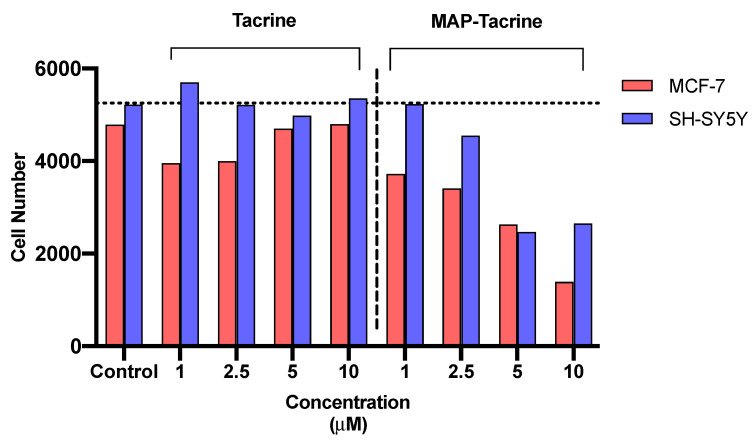
Cell count through Lionheart FX treatment of MCF-7 and SH-SY5Y cell lines with Tacrine and Tacrine-MAP (1, 2.5, 5, and 10 µM) after 24h incubation (*n* = 1).

A cell count analysis was also conducted and corroborated data from MTT assays for both cell lines. Hence, it was confirmed that, while Tacrine did not affect the number of cells in culture up to 10 μM, the MAP-Tacrine conjugate significantly decreased cell count (Figure 5), thus displaying strong toxicity to both types of cancer cells.

### 2.5. Determination of IC_50_ and Cell Count Analysis of Tacrine, MAP, and Tacrine-MAP against MCF-7

**Figure 6:**
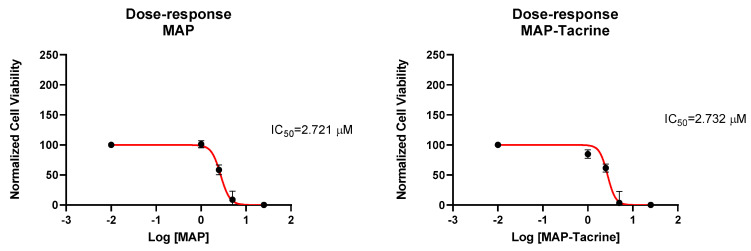
Dose-response curves (from triplicates) of MCF-7 cells viability with increasing concentrations (1, 2.5, 5, 10, 20, and 25 µM) of MAP (left plot) and MAP-Tacrine (right plot). IC50 values thus determined are shown next to the respective curves. The values represent mean ± SEM (*n* = 3).

**Figure 7:**
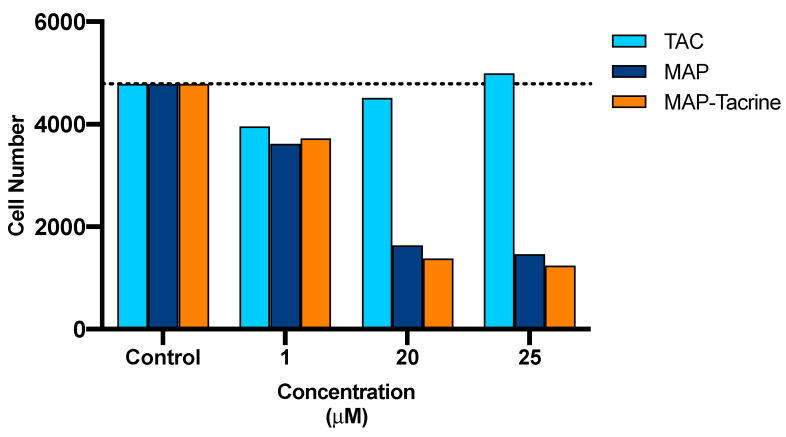
Cell count through Lionheart FX treatment of MCF-7 lines with Tacrine, MAP, and Tacrine-MAP (1, 20, and 25 µM) after 24 h incubation (*n* = 1).

The effect of coupling MAP to Tacrine on the intrinsic cytotoxic activity of the parent peptide was also assessed on MCF-7 cells. Dose-response curves are depicted in Figure 6 and show similar IC_50_ values of 2.721 µM and 2.732 µM for both MAP and MAP-Tacrine, respectively. Moreover, cell count analysis after treatment with increasing concentrations of Tacrine, MAP, and MAP-Tacrine, show that while Tacrine has no significant effects, both MAP and its conjugate cause a severe drop in cell count at 20 and 25 µM (Figure 7).

### 2.6. Morphological Changes Caused by Tacrine, MAP, and Tacrine-MAP on MCF-7 Cells

**Figure 8:**
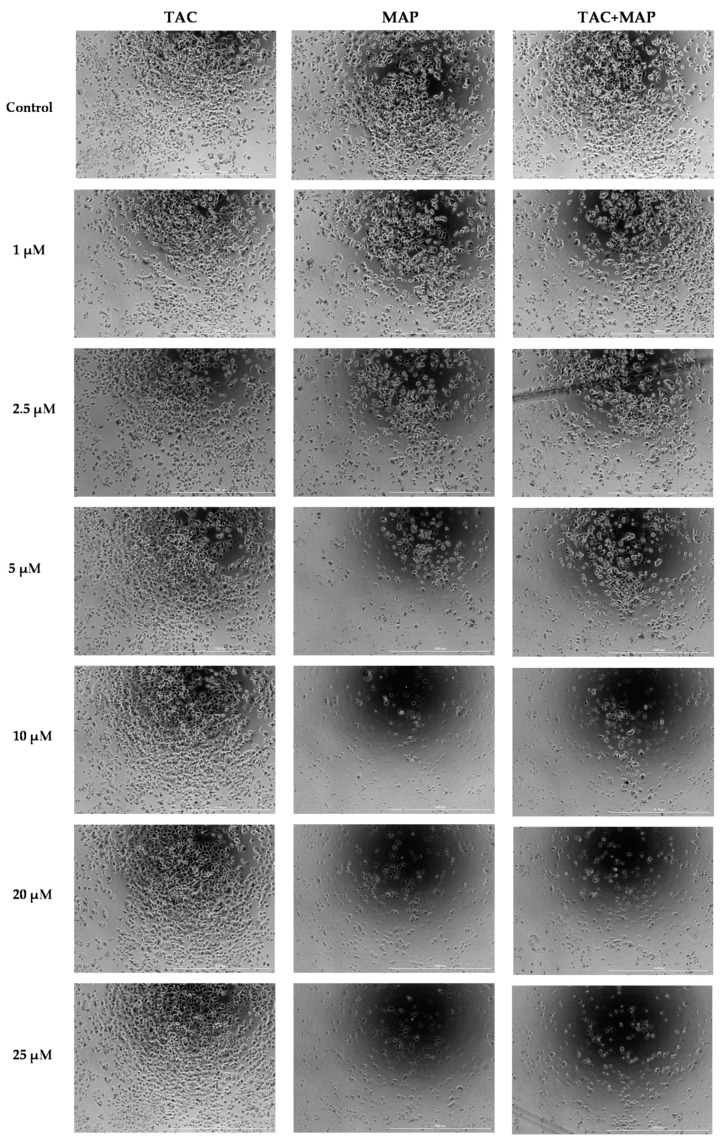
Micrographs (Lionheart FX of phase-contrast images) obtained for MCF-7 cells after a 24 h period of incubation with Tacrine, MAP, and MAP-Tacrine conjugate at different concentrations.

Morphological changes in MCF-7 cells after 24 h incubation with Tacrine, MAP, Tacrine-MAP at different concentrations were evaluated by Lionheart FX (Figure 8). No significant differences in cell morphology were observed between the control and Tacrine at all concentrations tested, as expected. In turn, MAP was confirmed to exert toxic effects on the cells, as significant differences are observed when comparing the effects of the peptide at 5, 10, 20, and 25 μM to control, including an increase of cells in suspension, loss of morphological features and cell death. Even at 2.5 μM, a slight increase of cells in suspension is already observed when compared to control. In the case of the MAP-Tacrine-conjugate, cell damage observed was similar to that caused by MAP alone, with substantial morphological changes and an increased level of cell death at higher concentrations (Figure 8).

### 2.7. Drug Interactions between Tacrine and MAP in the Conjugate

**Figure 9:**
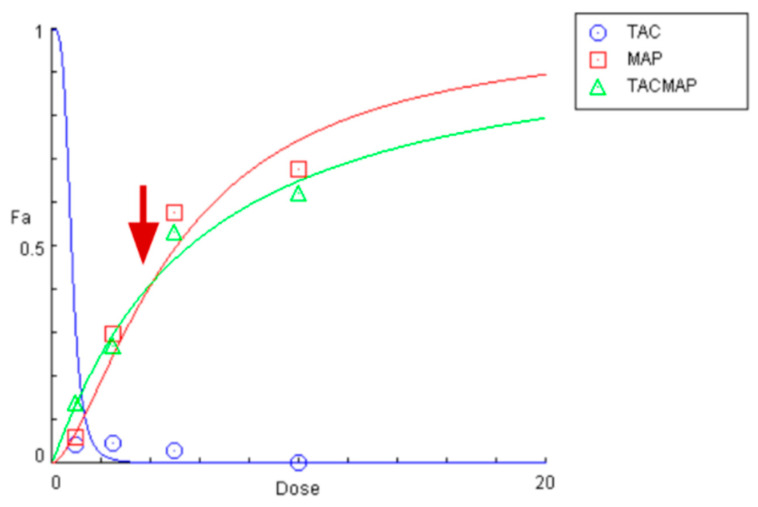
Dose-effect curves of Tacrine, MAP, and MAP-tacrine estimated by the unified theory using CompuSyn software. The dose was plotted on the *y*-axis as a function of effect level (Fa) on the *x*-axis to assess each drug behavior. Doses below the red arrow indicate better activity of Tacrine-MAP conjugate than MAP and tacrine alone.Experiments were conducted in triplicate (*n* = 3).

In Figure 9, we can observe the impact of synergism and antagonism in the MAP combination with Tacrine. Using our data, it is possible to confirm that we have a synergistic effect in decreasing cell viability for lower doses of drug and peptide and, an additive effect for higher doses. The fact that we observe synergism only in smaller concentrations, allows us to think that any secondary effects associated with peptides can be minimized in conjugates such as the one we report. In addition, the effect of MAP and Tacrine can be considered super-additive and demonstrate action that is above what is expected from their individual potencies and efficacies at a lower concentration

## 3. Concluding Remarks

The development of novel drugs for cancer therapy can be challenging, time-consuming, and expensive. As such, in order to overcome these hurdles, the combination and repurposing of drugs have been gaining prominence recently. Both strategies allow reducing overall costs and offer a fast-testing track for new therapeutic indications other than the original ones. In view of this and based on the previously reported anticancer potential of Tacrine, we explored how such potential might be enhanced by coupling this neuroactive drug to a CPP. To this end, we successfully synthesized both the selected CPP, MAP, and its Tacrine conjugate, formed via the well-known CuAAC click reaction. Both the conjugate and its parent building blocks were characterized by a toolbox of in vitro techniques, which allowed us to conclude that despite the conjugate no longer retaining the ability of the parent drug to cross the BBB or inhibit AChE, it displays significant cytotoxic action against two cancer cell lines, namely breast (MCF-7) and neuroblastoma (SH-SY5Y) cells. This cytotoxic action was coherently observed in different types of assays, including the assessment of cell counts and morphological changes. Relevantly, this same cytotoxic effect was observed for the MAP peptide alone, which, on the one hand, puts into question if Tacrine is really important for the effects observed, and on the other, highlights a cell destructive rather than a cell-penetrating role for the peptide. This highlights the potential use of MAP not as a classic CPP, but more as an antimicrobial peptide (AMP), able to destroy cell membranes even at low concentration levels, leading to fast cell death. Indeed, the behavior observed for both MAP and its Tacrine conjugate in this work closely resemble those reported in previous works focused on AMP, including examples where these AMP were found to exert selective membrane toxicity against malignant cells [17,24,25,26,27]. The observation of alterations on membrane integrity caused by MAP or its Tacrine conjugate has no precedent in the literature but, interestingly, comes into agreement with very recent reports highlighting the lytic character of CPP-drug conjugates comprising heterocyclic drug moieties [27,28].

## 4. Materials and Methods

### 4.1. MAP and Conjugate Synthesis

#### 4.1.1. General Peptide Synthesis Procedure

The peptide (C-terminal amide) was assembled by Fmoc/tBu SPPS methodologies. The resin was preconditioned for 15 min in *N*,*N*-dimethylformamide (DMF). The initial Fmoc deprotection step was carried out for 20 min using 20% piperidine in DMF. The C-terminal amino acid was then coupled to the deprotected Rink amide resin, using five molar equivalents (eq) of the Fmoc-protected amino acid in DMF, 5 eq of hexafluorophosphate benzotriazole tetramethyl uronium (HBTU) in DMF, and 10 eq of *N*-ethyl-*N*,*N*-diisopropylamine; this coupling step was carried out for 1 h. The remaining amino acids were sequentially coupled in the C → N direction by means of similar deprotection and coupling cycles. Following the completion of the sequence assembly, the peptide was released from the resin with concomitant removal of side-chain protecting groups, by a 2 h acidolysis at room temperature using a TFA-based cocktail containing triisopropysilane and water (95:2.5:2.5 *v/v/v*) as scavengers. The synthesis crude was obtained by precipitation using *tert*-butylmethyl ether (<0 °C).

#### 4.1.2. General Analysis Procedure

Peptide or conjugate analysis and purity degree were determined by HPLC, using a Hitachi-Merck Elite LaChrom system from Hitachi High-Technologies America (Schaumburg, IL, USA) equipped with a quaternary pump, a thermostatted automated sampler, a diode-array detector (DAD), and a reverse phase C18 Nucleodur gravity column (Macherey-Nagel Inc., Bethlehem, PA, USA) with 5 µm particle size and the dimensions 4 mm ID × 125 mm. The samples were solubilized in aqueous acetic acid (10% *v/v*) and eluted over a gradient of 100% solvent A (100% H_2_O with 0.05% (*v/v*) TFA) to reach 100% Solvent B (ACN with 0.05% (*v/v*) TFA) for 30 min, at a flow rate of 1 mL/min and detection at λ = 220 nm.

Synthetic crudes were purified by a preparative HPLC system (LaPrep Sigma VWR, Pennsylvania, USA), equipped with a UV detector, a quaternary LPG pump injection with a fractionation valve pump, and a reverse-phase C18 column (250 × 25 mm ID with 5 µM pore size, Merck, Darmstadt, Germany). The mobile phase was a gradient elution of acidified H_2_O (0.05% TFA)/ACN mixtures that are reported in the SI. Fractions containing the pure product (determined by HPLC) were pooled and freeze-dried.

MS analysis was performed on an LTQ Orbitrap™ XL hybrid mass spectrometer (Thermo Fischer Scientific, Bremen, Germany) controlled by LTQ Tune Plus 2.5.5 (Thermo Fischer Scientific, Bremen, Germany) and Xcalibur 2.1.0 (Thermo Fischer Scientific, Bremen, Germany). The electrospray ionization source settings were as follows: source voltage, 3.1 kV; the capillary temperature was 275 °C with a sheath gas flow rate at 40 and auxiliary gas flow rate at 10 (arbitrary unit as provided by the software settings). The capillary voltage was 36 V and the tube lens voltage 110 V.

The direct electrospray ionization-ion trap mass spectrometry (ESI-IT MS) analysis was performed on a Finnigan Surveyor LCQ DECA XP MAX mass spectrometer from ThermoElectron Corporation (Waltham, MA, USA), and the LC-MS analysis was performed on the same equipment, coupled to a Finnigan Surveyor HPLC, equipped with a DAD Plus Detector, an Autosampler Plus, and an LC Pump Plus. MALDI-TOF/TOF matrix-assisted desorption/ionization mass spectrometry was performed using a Bruker Daltonics UltrafleXtreme (Bruker, MA, USA).

MS data handling software (Xcalibur QualBrowser software, Thermo Fischer Scientific, Bremen, Germany) was used to obtain the confirmation of the synthetic peptides by their exact m/z value. All target products were obtained with correct mass spectral data and high chromatographic purity.

### 4.2. Cell Culture

#### 4.2.1. Materials

All plastic sterile material was obtained from Starstedt (Nümbrecht, Germany), except 48-well plates, 12-well plates, and tissue cultures flasks used in the permeability assays that were purchased from Corning Inc (Corning, New York, NY, USA), VWR (Radnor, PA, USA) and Orange Scientific (Braine-l’Alleud, Belgium), respectively. Trypsin-EDTA used for the cytotoxicity studies was acquired from Alfagene (Carcavelos, Portugal). Dulbecco’s Modified Eagle medium (DMEM), Dimethyl-sulfoxide (DMSO), and Fetal Bovine Serum (FBS) used in SH-SY5Y cells and MCF-7 cells were purchased from Merck (Kenilworth, NJ, USA). Endothelial Cell Growth Medium-2 (EBM-2) was acquired from Lonza (Basel, Switzerland). Regarding the cytotoxicity assays, Dulbecco’s Phosphate Buffered Saline (PBS) without calcium and magnesium, the solution of penicillin ( 10,000 U/mL) and streptomycin (10 mg/mL), the Hank’s Balanced Salt Solution (HBSS), and the 3-(4,5-dimethyl-2-thiazolyl)-2,5-diphenyl-2H-tetrazolium bromide (MTT) powder were purchased from Sigma-Aldrich, while other reagents from other assays were also acquired from there, including hydrocortisone, HEPES, ascorbic acid, basic fibroblast growth factor, the enzyme acetylcholinesterase (AChE from the electric eel), 5,5′-dithiobis-(2-nitrobenzoic acid), acetylcholine iodide, and Tacrine. For the permeability assays, FBS, penicillin, streptomycin, trypsin-EDTA, collagen I rat protein, HBSS, PBS, and chemically defined lipid concentrate were purchased from Gibco (Waltham, MA, USA). Triton X-100 was acquired from SpiChem (Gessate, Italy). The human immortalized endothelial cell line (hCMEC/D3) was purchased from Cedarlane (Hornby, ON, Canada). The BD Falcon^®^ cell culture inserts (transparent polyethylene terephthalate membrane) with a 0.4 μm pore size were from BD Biosciences (Franklin Lakes, NJ, USA).

#### 4.2.2. Cell Culture Conditions

The hCMEC/D3 cells were initially maintained in 25 cm^2^ culture flasks after thawing with complete growth medium [EBM-2 medium was supplemented with 5% FBS, bFGF (1 ng/mL), penicillin-streptomycin (final concentration of 100 U/mL and 0.1 mg/mL, respectively), chemically defined lipid concentration (1/100, *v/v*), ascorbic acid (5 μg/mL), hydrocortisone (1.4 μM), and HEPES (10 mM)] in an incubator at 37 °C and 5% CO_2_. After the first passage, the cells were maintained in 75 cm^2^ culture flasks. The number of cell passages used ranged from 35 to 41. Cell passaging by trypsinization was done every 3–4 days and the culture medium was replaced every two days.

MCF-7 cells and SH-SY5Y cells were maintained in a culture flask (25 cm^2^) with full growth medium DMEM supplemented with 10% FBS and penicillin and streptomycin (final concentration of 100 U/mL and 0.1 mg/mL, respectively) were kept in an incubator at 37 °C and 5% CO_2_. In the culture flask, the medium was changed at least twice a week, while trypsinization was done at least once a week. Trypsinization was made when there was more than 80% confluence of the monolayer and cell passages used were lower than 35.

### 4.3. Permeability Studies

For the permeability studies, the model was set with the insert (0.4 μm pore) being coated with rat tail collagen type I (50 μg/mL) for 1 h, to promote cell adherence and washed two times with PBS. The cells were seeded at a density of 2.5 × 10^4^ cells/cm^2^ on the AP side of the filter. The endothelial hCMEC/D3 cells were cultured in the insert for eight days at the conditions referred to (37 °C and 5% CO_2_). The culture medium was replaced every two days. The TEER was used to analyze the monolayer integrity and it was periodically determined using an endothelial voltammeter (World Precision Instruments, Sarasota, FL, USA). The resistance, expressed in Ω/cm^2^, was achieved by subtracting the value of an empty filter from the value of each measurement. The TEER values obtained throughout the maintenance of the cells were constant. On the day of the assays, 500 μL of Tacrine, MAP, and Tacrine-MAP diluted in HBSS (concentration of 40 μM for all compounds) was added to the upper compartment. All conditions were done in triplicate. Before and after adding the compound to the AP side, 750 μL of preheated HBSS were inserted in the lower compartment, to prevent tearing the membrane.

During the assay, the cells were kept at 37 °C in an orbital shaker incubator (100 rpm). 200 μL of samples were taken from the BL side at predetermined time points (15, 30, 45, 60, 90, 120, 180, and 240 min). To replace the taken volume, 200 μL of preheated HBSS was added. At every time interval before adding the samples, the TEER was measured in all conditions to detect changes in the monolayer confluence. The values of this electrical resistance are expressed as the variance in relation to the TEER value of the beginning of the assay and they are represented as mean ± SD. At the end of the experiment, samples of the AP side were also taken.

### 4.4. Cytotoxicity Assays

For cytotoxicity evaluation, MCF-7 cells were seeded in 48-well plates at a density of 25 × 10^4^ cells per well in a final volume of 250 µL of culture medium. Cells were given 24 h to attach before they were incubated with the compounds in the study. The cells were treated with different concentrations (1, 2.5, 5, 10, 20, and 25 µM) of Tacrine, MAP, and Tacrine-MAP for 24 h with a final volume per well of 250 μL of complete medium. H_2_O and DMSO were used in control, with the volume added being the one used for each concentration.

SH-SY5Y cells were seeded in 96 well-plate at a density of 18,000 cells per well at the final volume of 200 µL. Cells were given 24 h to attach before they were incubated with the compounds in the study. The cells were treated with different concentrations (1, 2.5, 5, and 10 µM) of Tacrine, MAP, and Tacrine-MAP for 24 h with a final volume per well of 200 μL of complete medium. H_2_O and DMSO were used in control, with the volume added being the one used for each concentration. After the period of incubation, the MTT reduction was performed in both cell lines.

#### MTT Reduction Assay

The MTT assay can be used to assess the cell’s metabolic activity [26]. MTT, a water-soluble compound, is reduced by the complex II of the mitochondrial respiratory chain to a water-insoluble blue formazan product. Only viable cells can metabolize MTT, so the metabolic activity is proportional to the quantity of formazans produced). After a 24 h incubation with the compounds in the study at 37 °C with 5% CO_2_, the cells were retrieved from the incubator and the medium was discarded. A previously warmed medium with MTT (0.5 mg/mL) was added (200 μL per well) and cells were maintained for 3 h at 37 °C and 5% CO_2_. Then the medium was aspirated again and DMSO (200 μL) was added to cause cellular lysis and solubilize the formazan crystals. The complete dissolution of the crystals was obtained by putting the plates on a shaking platform for 15 min, protected from light. The absorbance was read at 570 nm in a microplate reader (BioTeK Model Synergy HT, Winooski, VT, USA). Results were expressed as percentage of control cells.

### 4.5. Ellman’s Method

To assess if the chemical linkage of MAP to Tacrine interferes with the latter’s ability to inhibit the AChE, we chose Ellman’s method [29] introduced in 1961. This method is still widely used with some modifications [30]. Apart from its main function, this enzyme can efficiently hydrolyze a sulfur analogue of acetylcholine–acetylthiocholine. The products of this reaction are acetate and thiocholine, with the latter reacting with the highly reactive DNTB ion, generating a 5-thio-2-nitrobenzoate anion that has a yellow color and it can be detected by measuring the increase of absorbance at 412 nm. 3 mL of assay medium (phosphate buffer pH 7.4 + 150 µL DNTB 0.01 M + 30 µL AChE 2.0 units/mL) was put into cuvettes. The positive control was incubated at 37 °C for 15 min and then was added 150 µL of substrate (acetylthiocholine 0.01 M). For the experiments with Tacrine and the conjugate, the test compounds were added to the assay solution and incubated at the same temperature for the same period, adding 150 µL of substrate after the incubation. After the addition of substrate to the assay medium, it was measured the increase in absorbance at 412 nm for 30 s intervals for 5 min (300 s) at 37 °C.

The absorbance was measured in the JENWAY 6300 Spectrophotometer. All conditions were made in triplicates. The value of the initial absorbance was subtracted to the value of the absorbance at 300 s and that was considered as the biological activity.

### 4.6. Cell Morphology Visualization and Cell Count Analysis

After the treatments, cell morphology and growth were assessed by the contrast phase microscope Lionheart FX (Biotech, VT, USA) with the use of Gen5 software (Biotech, USA). The cell count of MCF-7 cells was assessed by Lionheart FX (Biotech, USA) using Gen5 software (Biotech, USA).

### 4.7. Analysis of Drug Interactions

To quantify the interaction of Tacrine and MAP in the conjugate, the dose-effect curves were estimated by the unified theory, introduced by Chou and Talalay [31] using CompuSyn software (ComboSyn, Inc., New York, NY, USA). The dose was plotted on the *y*-axis as a function of effect level (Fa) on the *x*-axis to assess the drugs’ behavior. Experiments were conducted in triplicate (*n* = 3) with three replications.

### 4.8. Statistical Analysis

Results are expressed in mean ± standard error of the mean (SEM). To assess whether the data was normal or not, the Student’s *t*-test was performed. If data distribution was normal an ordinary one-way ANOVA statistical analysis was executed, by the Student’s t-test. Statistical significance was accepted when *p* < 0.05. The GraphPad Prism 8 software (San Diego, CA, USA) was used to perform all statistical analysis.

## Supplementary Materials

Supplementary Materials can be found at https://www.mdpi.com/1422-0067/22/1/242/s1. Figure S1: Structure of MAP; Figure S2: Chromatogram of synthetic MAP, acquired with an HPLC system, with a C18 column, using ACN and acidified water (0.05% TFA) as eluent, in gradient mode (0–100%), for 30 min, at a flow rate of 1 mL/min and detection at λ = 220 nm; Figure S3: Mass spectrum (LC-ESI/Orbitrap MS, positive mode) of MAP; Figure S4: Chromatogram of synthetic crude of Pra-MAP (2), acquired with an HPLC system, with a C18 column, using ACN and acidified water (0.05% TFA) as eluent, in gradient mode (0–100%), for 30 min, at a flow rate of 1 mL/min and detection at λ = 220 nm; Figure S5: MALDI-TOF mass spectrometry of synthetic crude of Pra-MAP; Figure S6: Chemical equation translating the synthesis of N-chloroacetyl-tacrine; Figure S7: Mass spectrum (LC-ESI/Orbitrap MS, positive mode) of N-chloroacetyl-tacrine; Figure S8: ^1^H NMR spectrum of N-chloroacetyl-tacrine (400 MHz, CDCl_3_). δ(ppm), 8.52 (s, 1H), 8.00 (d, J = 8.4 Hz, 1H), 7.75 (d, J = 8.4 Hz, 1H), 7.67–7.57 (m, 1H), 7.51–7.41 (m, 1H), 4.34 (s, 2H), 3.13 (t, J = 6.5Hz, 2H), 2.80 (t, J = 6.4 Hz, 2H), 2.01–1.90 (m, 2H), 1.89–1.81 (m, 2H).; Figure S9: Chemical equation translating the synthesis of N-azidoacetyl-tacrine (1); Figure S10: Mass spectrum (LC-ESI/Orbitrap MS, positive mode) of N-azidoacetyl-tacrine (1); Figure S11: ^1^H NMR of N-azidoacetyl-Tacrine (400 MHz, CD3OD). δ(ppm), 7.92 (dd, J = 14.4, 8.4 Hz, 2H), 7.72–7.66 (m, 1H), 7.57–7.51 (m, 1H), 4.25 (s, 2H), 3.12 (t, J = 6.5 Hz, 2H), 2.84 (t, J = 6.5 Hz, 2H), 2.01–1.94 (m, 2H), 1.92–1.84 (m, 2H); Figure S12: ^13^CNMR of N-azidoacetyl-Tacrine (101 MHz, CD_3_OD). δ(ppm), 169.11 (C=O), 161.01 (C), 146.98 (C), 140.99 (C), 130.63 (CH), 129.61 (CH), 127.91 (CH), 127.54 (CH), 125.47 (CH), 123.85 (CH), 52.99 (CH_2_), 34.08 (CH_2_), 26.18 (CH_2_), 23.43 (CH_2_), 23.22 (CH_2_). Figure S13: Chemical equation of the synthesis (top) and structure (bottom) of the MAP-Tacrine conjugate 3. Figure S14: Chromatogram of synthetic MAP-Tacrine conjugate 3, acquired with an HPLC system, with a C18 column, using ACN and acidified water (0.05% TFA) as eluent, in gradient mode (0–100%), for 30 min, at a flow rate of 1 mL/min and detection at λ = 220 nm. Figure S15: Mass spectrum (LC-ESI/Orbitrap MS, positive mode) of the Tacrine-MAP conjugate. Figure S16: Results are expressed in mean ± SD, *n* = 3 independent experiments. TEER measurements for all compounds’ overall time-points. Two-way ANOVA test followed by Tukey’s post hoc test. No significant differences were observed; Table S1: Initial and final values of ΔTEER. Results represented as mean ± SD, *n* = 3 independent experiments.

## Abbreviations

5-FU	5-Fluorouracil
AChE	Acetylcholinesterase
ACN	Acetonitrile
AD	Alzheimer Disease
AMP	Antimicrobial peptide
BBB	Blood brain barrier
CPP	cell penetrating peptide
CuAAC	Copper-catalyzed azide−alkyne coupling
DAD	Diode-array detector
DIEA	Diisopropylethylamine
DMF	N,N-dimethylformamide
ESI-IT MS	Electrospray ionization-ion trap mass spectrometry
FDA	Food and Drug Administration
HOBt	1-hydroxybenzotriazole
HPLC	High performance liquid chromatography
LC-MS	Liquid chromatography–mass spectrometry
MALDI-TOF/TOF	Matrix assisted desorption/ionization mass spectrometry
MAP	model amphipathic peptide
MTT	3-(4,5-dimethyl-2-thiazolyl)-2,5-diphenyl-2H-tetrazolium bromide
MW	Microwave
NaAsc	Sodium ascorbate
SD	Standard deviation
SEM	Standard error of the mean
SPPS	solid-phase peptide synthesis
TEER	Trans-epithelial electrical resistance
TFA	Trifluoroacetic acid
