# Supplementary material for: Model Amphipathic Peptide Coupled with Tacrine to Improve Its Antiproliferative Activity
Source: Int J Mol Sci. 2020 Dec 29;22(1):242. doi: 10.3390/ijms22010242 (PMC7795729; doi:10.3390/ijms22010242)
Supplement: Supplementary file 1 [file ijms-22-00242-s001.pdf]

Supplementary Information

Model amphipathic peptide coupled with Tacrine to improve its antiproliferative activity

Sara Silva[1,2,3], Cláudia Alves[4], Diana Duarte[1,2], Ana Costa[5,6], Bruno Sarmento[5,6,7,8], António J. Almeida[3], Paula Gomes[4] and Nuno Vale[1,9,]**

[1] OncoPharma Research Group, Center for Health Technology and Services Research (CINTESIS), Rua Dr. Plácido da Costa, 4200-450 Porto, Portugal
[2] Faculty of Pharmacy, University of Porto, Rua de Jorge Viterbo Ferreira, 228, 4050-313 Porto, Portugal
[3] Research Institute for Medicines (iMed.ULisboa), Faculty of Pharmacy, University of Lisbon, Avenida Professor Gama Pinto, 1649-003 Lisboa, Portugal
[4] LAQV-REQUIMTE, Departamento de Química e Bioquímica, Faculdade de Ciências, Universidade do Porto, Rua do Campo Alegre, 687, 4169-007 Porto, Portugal
[5] INEB, Instituto de Engenharia Biomédica, University of Porto, Rua Alfredo Allen, 208, 4200-135 Porto
[6] i3S, Instituto de Investigação e Inovação em Saúde, University of Porto, Rua Alfredo Allen, 208, 4200-135 Porto, Portugal
[7] CESPU, Instituto de Investigação e Formação Avançada em Ciências e Tecnologias da Saúde & Instituto Universitário de Ciências da Saúde, Rua Central de Gandra, 1317,4585-116, Gandra, Portugal
[8] School of Pharmacy, Queen's University Belfast, Medical Biology Centre, Belfast, UK
[9] Faculty of Medicine, University of Porto, Al. Prof. Hernâni Monteiro, 4200-319 Porto, Portugal

1. Chemical synthesis

1.1. Solid-phase synthesis of MAP

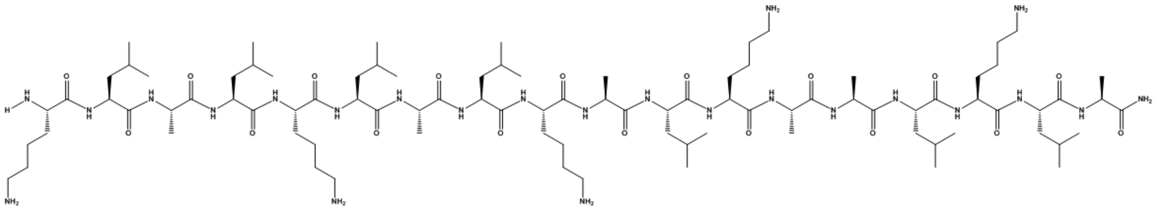

M= 1876.50 g/mol

Figure S1. Structure of MAP.

- Solid support: resin Rink Amide (0.38 mmol/g)
- Synthesis scale: 0.2 mmol
- Protection scheme: orthogonal (Fmoc/tBu)
- Pre-activation: 10 eq. DIEA, 5 eq. amino acidin5 mL DMF
- Coupling agent: 5 eq. HBTU
- Deprotection: 5 mL 20% piperidine in DMF
- Cleavage: 95% TFA + 2.5% H_2O + 2.5 %TIS, 1 mL/100 mg of peptidyl-resin, 2 h, room temperature
- Purification by preparative HPLC (20-50% acetonitrile, ACN) to a final 100% purity degree
- m (pure peptide) = 16.0 mg

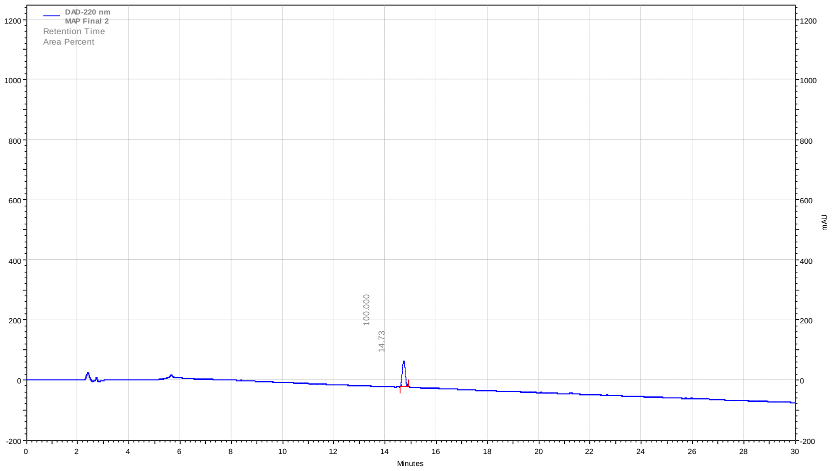

Figure S2. Chromatogram of synthetic MAP, acquired with a HPLC system, with a C18 column, using ACN and acidified water (0.05% TFA) as eluent, in gradient mode (0 – 100%), for 30 minutes, at a flow rate of 1 mL/min and detection at λ = 220 nm.

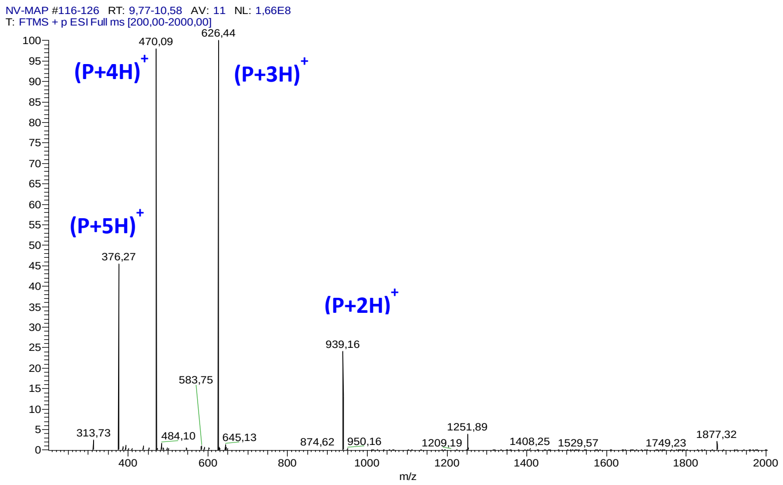

Figure S3. Mass spectrum (LC-ESI/Orbitrap MS, positive mode) of MAP.

1.2. Solid-phase synthesis of Pra-MAP (2)

- Solid support: resin Rink Amide (0.38 mmol/g)
- Synthesis scale: 0.2 mmol
- Protection scheme: orthogonal (Fmoc/tBu)
- Pre-activation: 10 eq. DIEA, 5 eq. amino acid in 5 mL DMF
- Coupling agent: 5 eq. HBTU
- Deprotection: 5 mL 20% piperidine in DMF
- Cleavage: 95% TFA + 2.5% H_2O + 2.5% TIS, 1 mL/100 mg of peptidyl-resin, 2 h, room temperature (only a small amount of peptidyl-resin was cleaved to allow HPLC and MS analysis of the functionalized peptide)
- Purification: not done (the Pra-MAP served as a reaction intermediary)
- Purity (crude): 80.4%
- m (product)= non taken

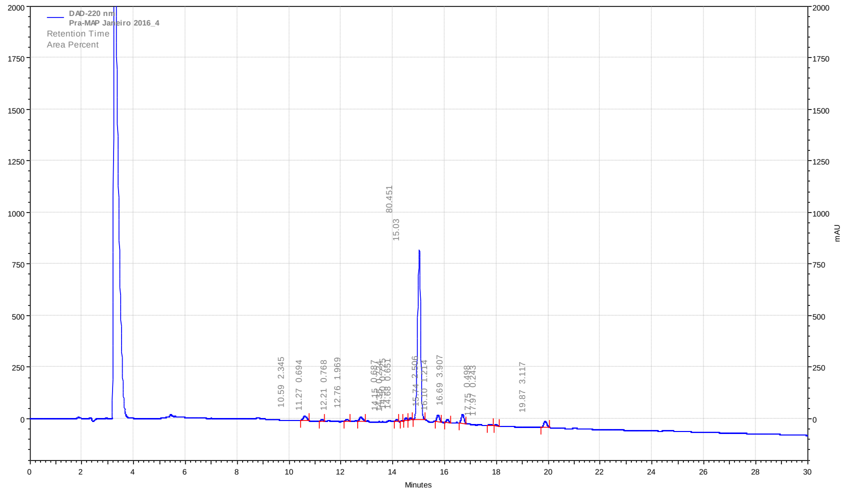

Figure S4. Chromatogram of synthetic crude of Pra-MAP (**2**), acquired with a HPLC system, with a C18 column, using ACN and acidified water (0.05% TFA) as eluent, in gradient mode (0 – 100%), for 30 minutes, at a flow rate of 1 mL/min and detection at λ = 220 nm.

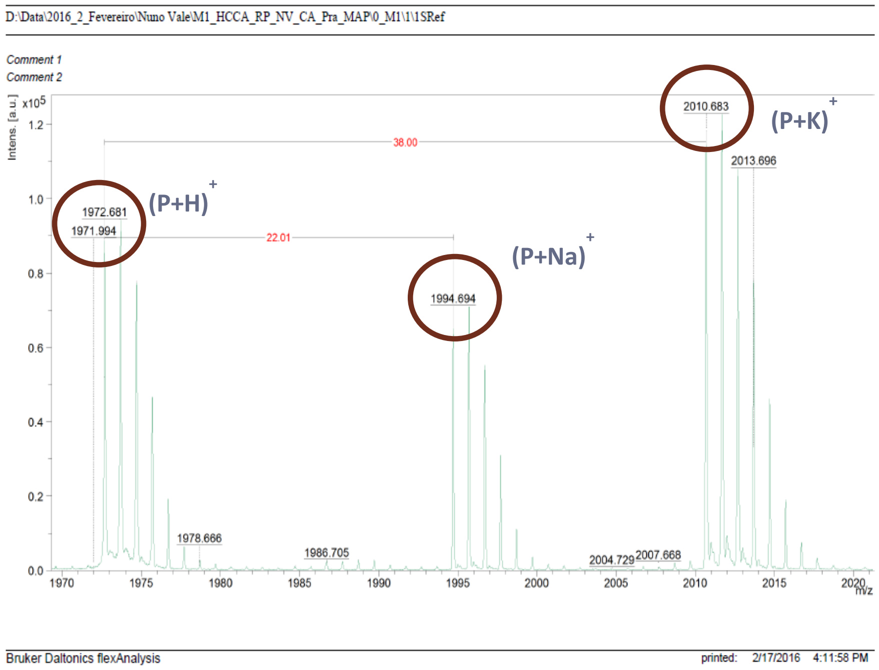

Figure S5. MALDI-TOF mass spectrometry of synthetic crude of Pra-MAP.

1.3. Synthesis of *N*-chloroacetyl-tacrine

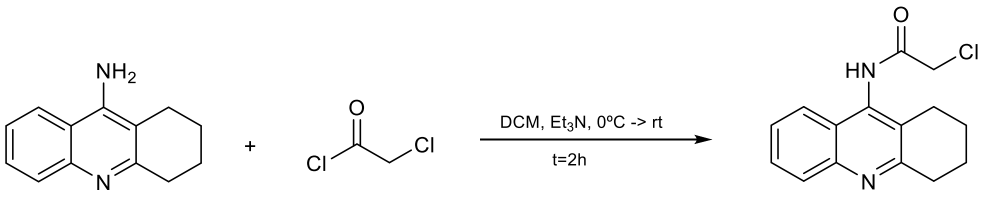

Tacrine
(anhydrous basis)

N-chloroacetyl-tacrine

Figure S6. Chemical equation translating the synthesis of *N*-chloroacetyl-tacrine.

- Tacrine pre-activation: 20 min stirring with 2 eq. NEt_3, in DCM (ice bath)
- m(Tacrine)= 200 mg (limiting reagent)
- Dropwise addition of 1.25 eq. chloroacethyl chloride
- 70 mL of solvent: dichloromethane (DCM)
- Reaction time: 2.5 h
- Work-up: liquid-liquid extractions with DCM + washes with brine
- Purification: silica-gel column, using DCM/Methanol 100:1 (v/v)
- Aspect: brownish solid
- m (product) = 65.4 mg
- η = 27.5 %

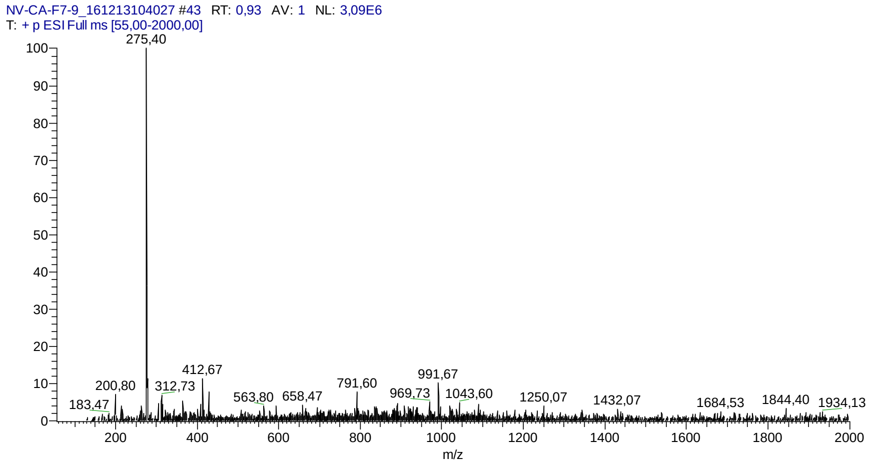

Figure S7. Mass spectrum (LC-ESI/Orbitrap MS, positive mode) of *N*-chloroacetyl-tacrine.

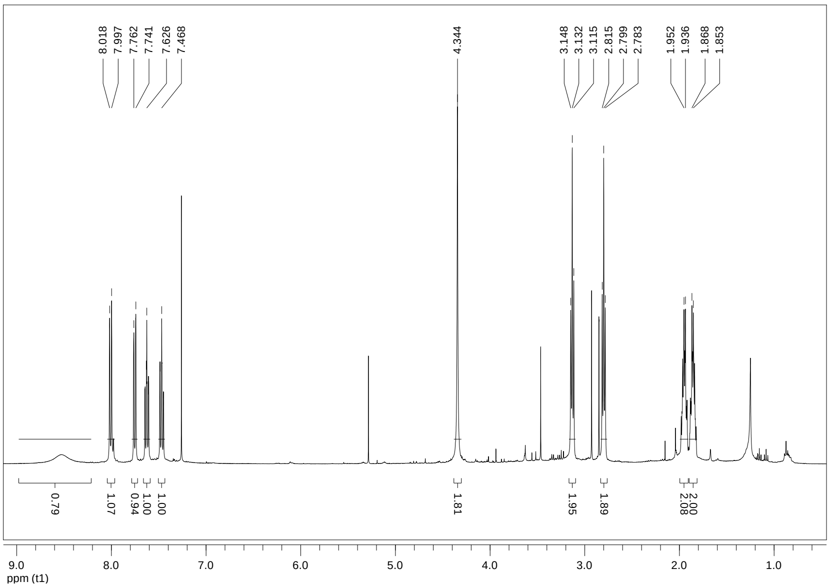

Figure S8. ^{1}H NMR spectrum of *N*-chloroacetyl-tacrine (400 MHz, $CDCl_3$).δ(ppm), 8.52 (s, 1H), 8.00 (d, *J* = 8.4 Hz, 1H), 7.75 (d, *J* = 8.4 Hz, 1H), 7.67 – 7.57 (m, 1H), 7.51 – 7.41 (m, 1H), 4.34 (s, 2H), 3.13 (t, *J* = 6.5Hz, 2H), 2.80 (t, *J* = 6.4 Hz, 2H), 2.01 – 1.90 (m, 2H), 1.89 – 1.81 (m, 2H).

1.4. Synthesis of *N*-azidoacetyl-tacrine (1)

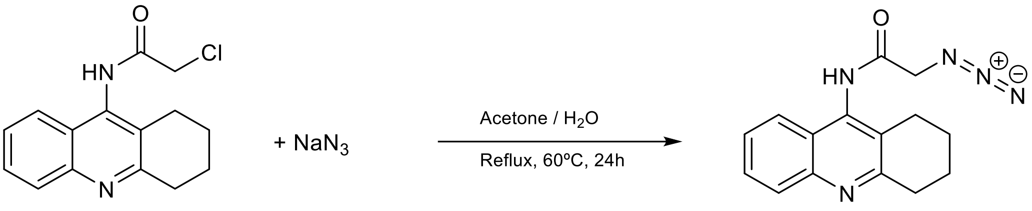

N-chloroacetyl-tacrine

N-azidoacetyl-tacrine (1)

Figure S9. Chemical equation translating the synthesis of *N*-azidoacetyl-tacrine (**1**).

- m (*N*-chloroacetyl-tacrine)= 18.4 mg (limiting reagent)
- Solvent: refluxing (60°C) acetone (5 mL) + H_2O (2.5 mL)
- Sodium azide (excess) – 5 eq.
- Reaction time: overnight
- Treatment: liquid-liquid extractions with DCM + H_2O
- Aspect: Orange brownish solid
- m (N-azidoacetyl-tacrine) = 20.2 mg
- η = 100 %

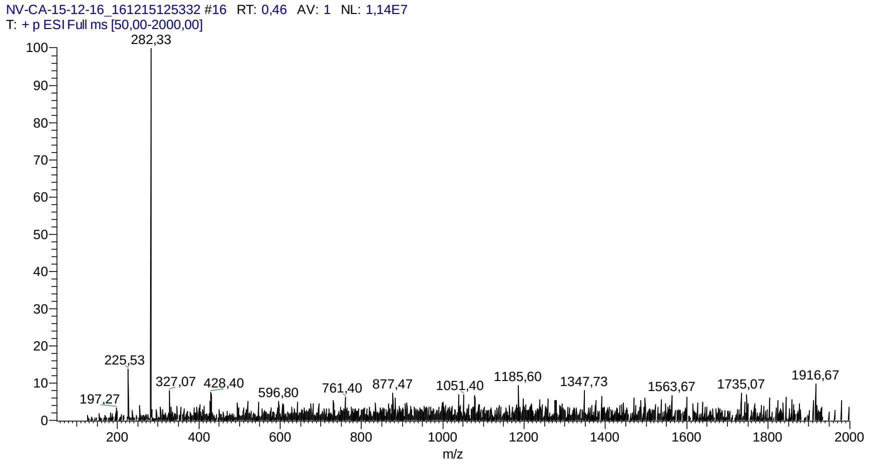

Figure S10. Mass spectrum (LC-ESI/Orbitrap MS, positive mode) of N-azidoacetyl-tacrine (**1**).

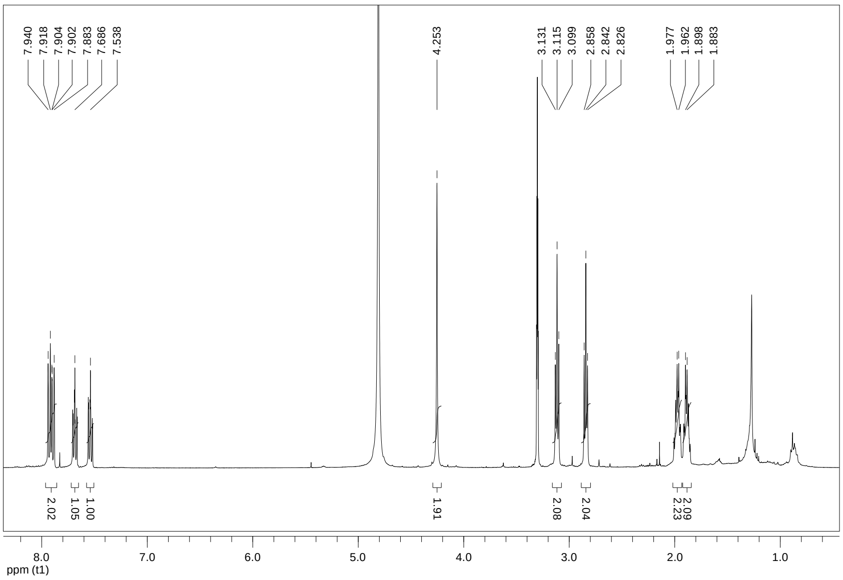

Figure S11. ^{1}H NMR of N-azidoacetyl-Tacrine (400 MHz, CD_3OD). δ(ppm),7.92 (dd, *J* = 14.4, 8.4 Hz, 2H), 7.72 – 7.66 (m, 1H), 7.57 – 7.51 (m, 1H), 4.25 (s, 2H), 3.12 (t, *J* = 6.5 Hz, 2H), 2.84 (t, *J* = 6.5 Hz, 2H), 2.01 – 1.94 (m, 2H), 1.92 – 1.84 (m, 2H).

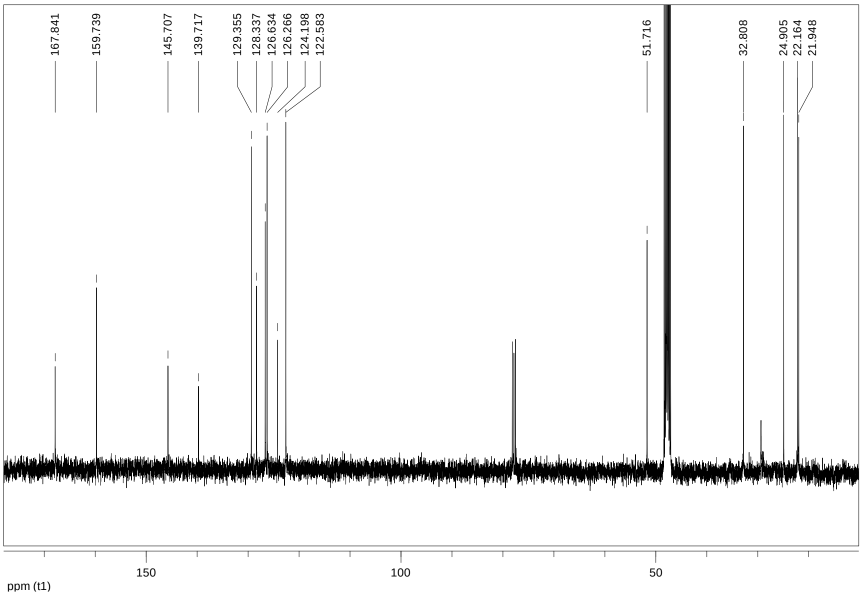

Figure S12. ^{13}C NMR of N-azidoacetyl-Tacrine (101 MHz, CD_3OD).δ(ppm), 169.11 (C=O), 161.01 (C), 146.98 (C), 140.99 (C), 130.63 (CH), 129.61 (CH), 127.91 (CH), 127.54 (CH), 125.47 (CH), 123.85 (CH), 52.99 (CH_2), 34.08 (CH_2), 26.18 (CH_2), 23.43 (CH_2), 23.22 (CH_2).

1.5. Synthesis of the MAP-Tacrine conjugate (3)

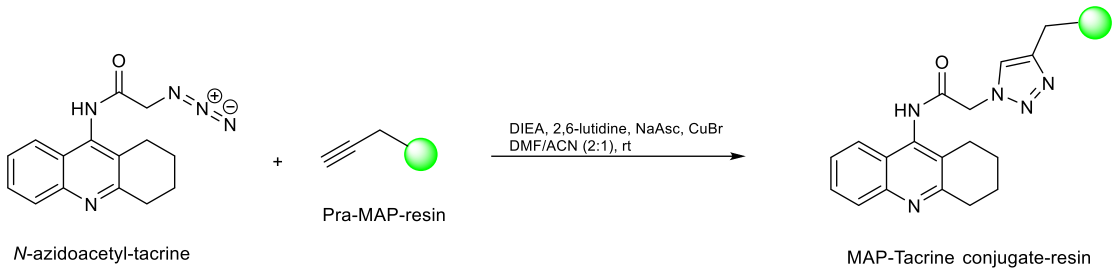

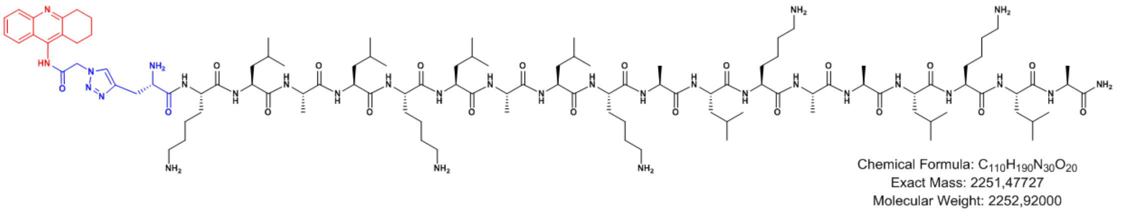

Figure S13. Chemical equation of the synthesis (top), and structure (bottom) of the MAP-Tacrine conjugate **3**.

- Solid support: resin Rink Amide (0.38 mmol/g)
- Synthesis scale: 0.2 mmol
- 1 eq. *N*-azidoacetyl-tacrine, 10 eq. DIEA, 10 eq. 2,6-lutidine, 1 eq. NaAsc, 1 eq. CuBr
- Solvent: 3 mL DMF + ACN (2:1)
- Reaction time: 40 h
- Work-up: resin wash (filtration) with H_2O, Methanol, DMF and DCM (3× each)
- Cleavage: 95% TFA + 2.5% H_2O + 2.5% TIS, 1 mL/100 mg of peptidyl-resin, 2 h, room temperature
- Purification by preparative HPLC (20-50% ACN) to a final 99.6% purity degree
- m (pure product) = 54.6 mg

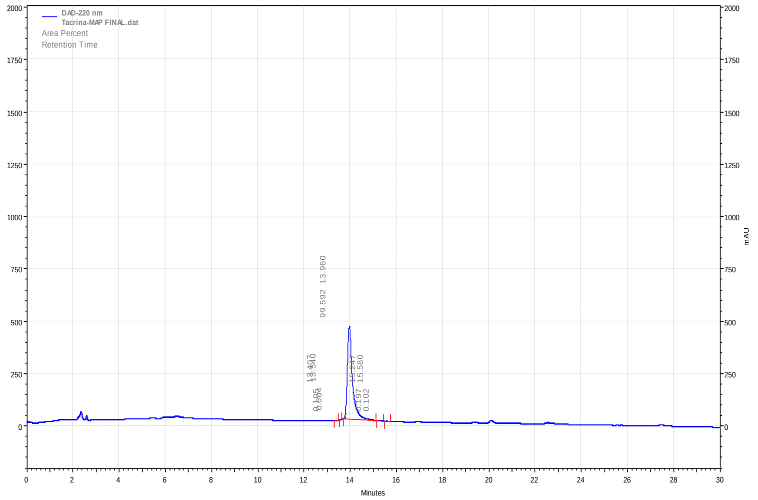

Figure S14. Chromatogram of synthetic MAP-Tacrine conjugate **3**, acquired with a HPLC system, with a C18 column, using ACN and acidified water (0.05% TFA) as eluent, in gradient mode (0 – 100%), for 30 minutes, at a flow rate of 1 mL/min and detection at λ = 220 nm.

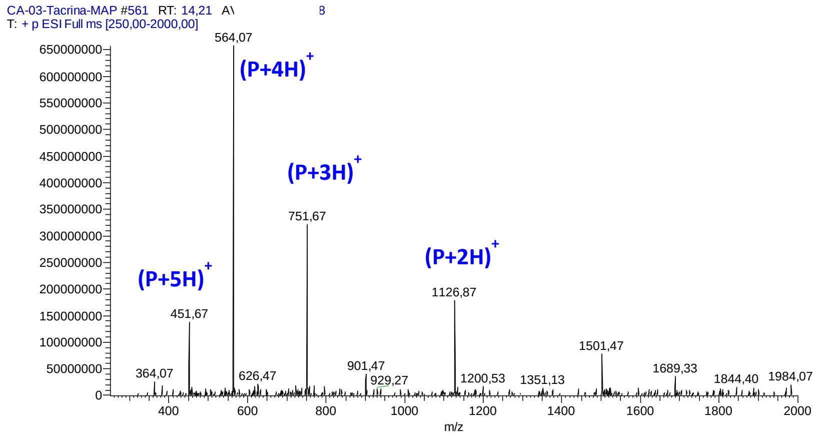

Figure S15. Mass spectrum (LC-ESI/Orbitrap MS, positive mode) of the Tacrine-MAP conjugate.

2. BBB permeability studies

Table S1 - Initial and final values of ΔTEER. Results represented as mean ± SD, n=3 independent experiments.

	ΔTEER at t=0 min	ΔTEER at t=240 min
Tacrine	0 ± 27	-36 ± 27
MAP	0 ± 49	-49 ± 6
Conjugate	0 ± 21	-29 ± 33

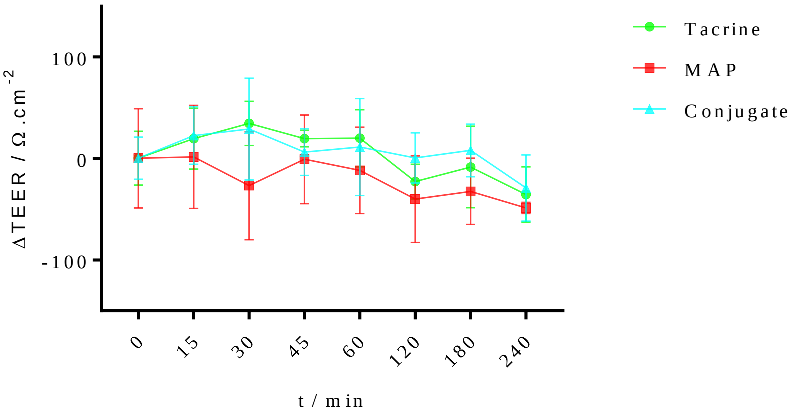

Figure S16. Results are expressed in mean ± SD, n=3 independent experiments. TEER measurements for all compounds over all time-points. Two-way ANOVA test followed by the Tukey's post hoc test. No significant differences were observed.
